# Wet Ball Milling Synthesis of Iron Oxide Nanoparticles for High-Efficiency, Controllable Magnetic Hyperthermia in Ovarian Cancer

**DOI:** 10.1002/admt.70960

**Published:** 2026-03-27

**Authors:** Shahriar Mostufa, Bahareh Rezaei, Md Shahriar, Karla Mercedes Paz González, Anil Kumar, Changxue Xu, Yun Suk Eo, Ioannis H. Karampelas, Jenifer Gómez-Pastora, Rui He, Kai Wu

**Affiliations:** 1 Department of Electrical and Computer Engineering, Texas Tech University, Lubbock, Texas, USA; 2 Department of Industrial, Manufacturing, and Systems Engineering, Texas Tech University, Lubbock, Texas, USA; 3 Department of Chemical Engineering, Texas Tech University, Lubbock, Texas, USA; 4 Department of Physics and Astronomy, Texas Tech University, Lubbock, Texas, USA; 5 Nemak USA, Inc., Sheboygan, Wisconsin, USA

## Abstract

Magnetic nanoparticles (MNPs) are attracting increasing attention for applications in energy, environment, and biomedicine. Among all MNP synthesis methods, ball milling is a cost-effective route for producing large quantities of MNPs at low cost. This work aims to investigate the magnetic hyperthermia performance of MNPs synthesized through a mechanochemical ball milling approach. Herein, we first varied the milling conditions and thoroughly characterized the physical properties of the produced MNPs; later, their hyperthermia performance was studied under different alternating magnetic fields (AMF). We report that the MNPs, after ball milling for up to 55 h at 200 rpm, show a higher magnetite phase with an average hydrodynamic size of ~270 nm and irregular morphology. These MNPs were subjected to clinically safe AMF (30 mT, 101.5 kHz), yielding a maximum temperature rise of ~50 °C, including in the SKOV3 cancer cell medium. Additionally, to enable controlled heating and avoid unintended damage to healthy tissues, we applied pulsed AMFs, achieving a temperature of ~30 °C. Lastly, the cellular uptake, colloidal stability, and biocompatibility tests were conducted on suspensions. This study reports a straightforward, cost-effective, large-scale synthesis route for MNPs and highlights their effective hyperthermia performance, showcasing their potential for future safer tumor treatment.

## 1 | Introduction

Magnetic nanoparticles (MNPs) have gained considerable attention for a wide range of applications, including the energy industry, environmental engineering, biomedical engineering, etc. For biomedical applications focusing on specific therapeutic purposes, MNPs are used as carriers for drug delivery [1], tracers for magnetic particle imaging (MPI) [2–5], contrast agents for magnetic resonance imaging (MRI) [6–9], and heating sources for magnetic hyperthermia [10–13]. In magnetic hyperthermia, to trigger tumor cell apoptosis, well-controlled, localized heating is induced by subjecting MNPs to an alternating magnetic field (AMF) [14–16]. The heat is produced via two mechanisms: one is the hysteresis loss, and the other is the relaxation loss (via Néel and Brownian relaxations) [11, 17]. This heat generation process from MNPs depends on several factors, such as intrinsic magnetic properties of the material, including saturatio magnetization and anisotropy, as well as extrinsic physicochemical properties such as size, shape, surface coating, etc. For superparamagnetic nanoparticles, heating primarily arises from Néel and Brownian relaxations, whereas for larger single-domain MNPs, hysteresis losses contribute significantly to heat generation [11]. The ideal temperature to kill cancer cells with minor injuries to the surrounding healthy cells has been reported to be between 42 °C and 46 °C [18–20]. Magnetite (Fe_3_O_4_) MNPs are among the most popular candidates for hyperthermia due to their high biocompatibility, environmental friendliness, and low cost.

For the synthesis of MNPs, various chemical methods are used, such as co-precipitation [21], hydrothermal [22], sol-gel [23], thermal decompositions [24], etc. Although chemical methods offer greater control over the size and shape of nanoparticles, the nanoparticle yields per batch are low. Mechanochemical-based ball milling presents several advantages, such as better reproducibility, scalability, and high product homogeneity [25]. This high-energy ball milling process generates MNPs from solid precursors without the need for organic solvents or high temperatures, and it also facilitates large-scale synthesis in cost-effective ways [26–28]. It is commonly used to produce MNPs, with milling parameters significantly influencing the structural properties of the resulting particles. Therefore, in this work, we synthesized MNPs using the ball milling method under varying conditions and evaluated their hyperthermia performance.

The in vivo applications of magnetic hyperthermia require that the product of the AMF amplitude and frequency adhere to the clinical safety threshold of 5 × 10^9^ A/m/s (Hergt–Dutz limit) [29, 30]. Therefore, the MNPs must generate enough heat under these constrained clinical safety conditions (*H* × *f*). To address this, researchers have proposed different methods to demonstrate the heat generation efficiency of nanoparticles by altering their physical properties within safety limits. For instance, Das et al. [31] reported Ag/Fe_3_O_4_ nanoflower-shaped MNPs with boosted magnetic hyperthermia performance (under an AMF of 80 mT and 310 kHz). When combined with a photothermal method, these MNPs generated heat up to nearly 70 °C. Sugumaran et al. [32] designed iron oxide-based nanorods to introduce shape anisotropies, thus affecting the hysteresis loop and enhancing hyperthermia heating performance (under an AMF of 90 mT and 100 kHz–1 MHz). With this nanorod design, they increased the SAR tremendously to 4,214 W/g. Also, Burnham et al. [33] reported the magnetic hyperthermia performance of MNPs prepared by the ball milling method with oleic acid precursors, under an AMF of 282 kHz; they achieved a maximum temperature change of 40 °C and SAR of 11.08 W/g. Pefanis et al. [34] conducted extensive studies on the impact of pulsed AMF on magnetic hyperthermia performance, where they varied the duty cycle (ON/OFF time) to demonstrate controlled heating from MNPs. Pulsed hyperthermia has several benefits over continuous hyperthermia [35]. For instance, it provides more control and flexibility over the temperature, and pulsing induces semi-adiabatic temperature growth at each pulse’s starting point. Additionally, depending on the therapeutic heating required, the pulse duty cycle can be varied to minimize the heating of surrounding healthy tissues. Thus, in this work, we additionally investigated the controlled hyperthermia of our MNPs by varying the AMF pulse widths. For future in vivo applications, the cellular uptake of the MNPs is crucial in enhancing the magnetic hyperthermia-based treatment, as it enables the intracellular nanoheating at low nanoparticle doses [36, 37]. Thus, we also characterized the biocompatibility and cellular uptake of our ball-milled MNPs by incubating them with SKOV3 ovarian cancer cells.

In this work, we have systematically studied the magnetocrystalline structures of ball-milled MNPs derived from Iron (Fe) powder and fine-tuned the ball-milling conditions to obtain MNPs capable of generating the maximum heat. This work highlights the magnetic hyperthermia performance of these synthesized ball-milled MNPs and demonstrates their cellular uptake, colloidal stability, biocompatibility, and hyperthermia performance in SKOV3 ovarian cancer cells medium. We have also varied the AMF field amplitude and frequency to study the effect of relaxation dynamics of these ball-milled MNPs on heating performance. In the following sections, we describe the details of the ball milling conditions and the materials employed (Section 2). Section 3 presents our main results and discussion, beginning with an analysis of the magnetocrystalline structure, morphology, particle size, and magnetic properties of the MNPs (3.1). This is followed by a detailed assessment of their magnetic hyperthermia performance (3.2), including the effects of field strength and frequency (3.3), pulsed AMF–based temperature regulation (3.4), and the influence of various surrounding media (3.5). Finally, we extend our investigation to study the cellular uptake, biocompatibility, and hyperthermia performance of the MNPs on SKOV3 ovarian cancer cells (3.6), which is of paramount importance for the clinical applications of these materials. Section 4 concludes with a summary of our findings on the physicochemical, hyperthermia, colloidal stability, cellular uptake, and biocompatibility characterizations of MNPs prepared by ball-milling methods, including an outlook for future use of these MNPs in other biomedical applications.

## 2 | Materials and Methods

### 2.1 | High-Energy Ball Milling

**FIGURE 1 |:**
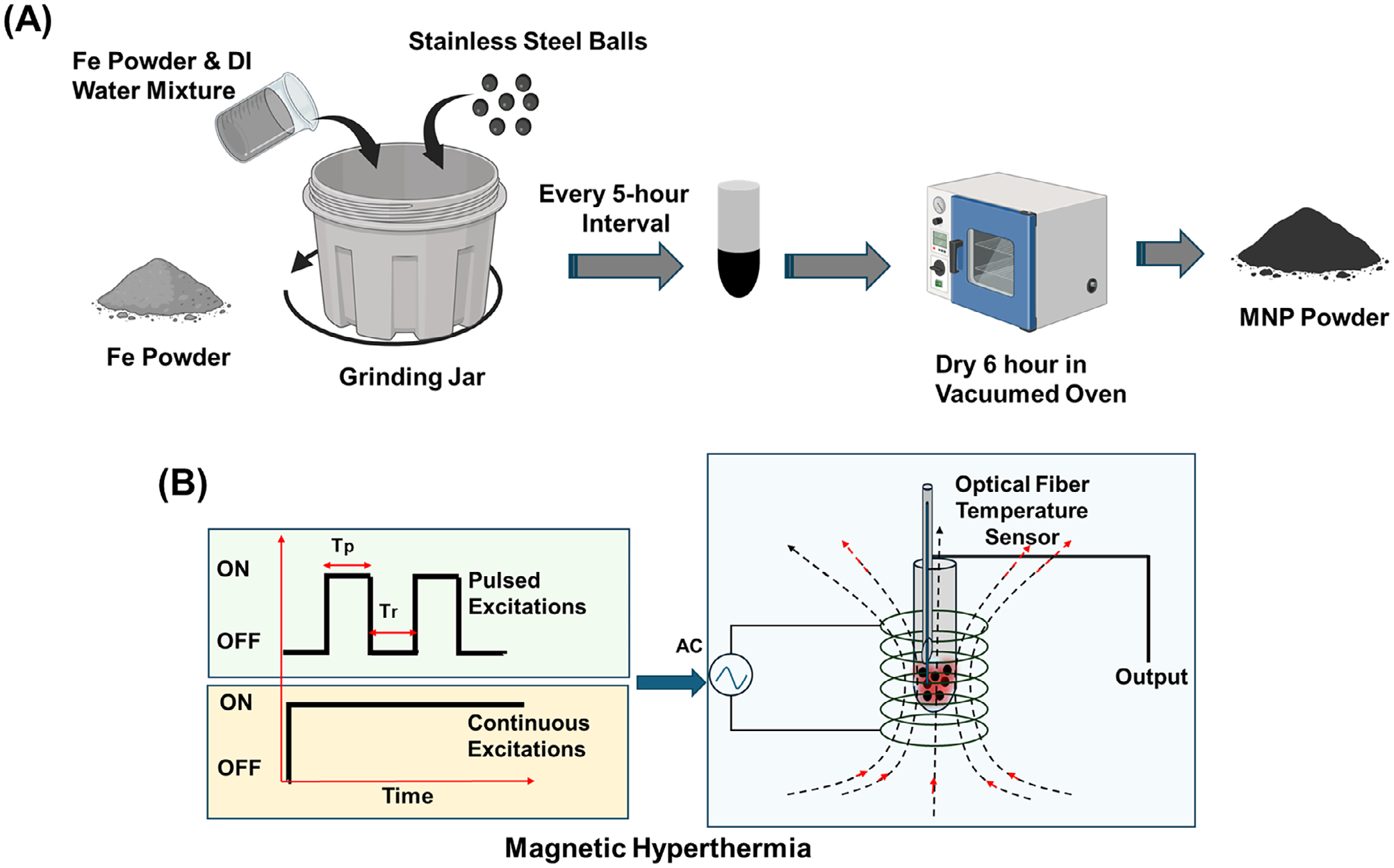
(A) Schematic representation of the mechanochemical synthesis of MNPs using a high-energy planetary ball mill. Figure partially drawn using BioRender. (B) Schematic of the magnetic hyperthermia experimental setup based on the calorimetric method.

The high-energy ball milling process was initiated using a 500 mL stainless steel jar containing stainless steel balls with an average diameter of 3 mm. The total weight of the balls was 700 g. As precursors, 36 g of high-purity iron (Fe) powder (≥99.9%, particle size <10 μm, CAS7439896, Sigma–Aldrich) and 72 mL of deionized (DI) water were used. This resulted in a ball-to-powder weight ratio of approximately 19.4:1. The combined weight of the grinding jar with all contents was around 5100 g. Ball milling was carried out using a PM-100 planetary ball miller (Retsch company) operating at 200 rpm in one-directional rotation. The milling was run in intervals of 5 h (cooled for 10 min after every 2.5 h), and samples were collected from the grinding jar after each 5 h interval. Milling continued for a total duration of 55 h because we had already achieved the desired heating performance with this milling time. Additionally, 20 mL of DI water was added to the jar after every 5 h interval to prevent the mixture from drying out. Figure 1A illustrates the schematics of our ball milling synthesis process and subsequent dry heating to obtain the MNP powder.

### 2.2 | Characterization of Ball-Milled MNPs

After sample collection, the nanoparticles were dried using a vacuum oven for around 6 h to obtain the final powder form. X-ray diffraction (XRD) measurements were performed on all samples using a Rigaku MiniFlex 6G diffractometer with a 1.25° slit size and Cu-K*α* radiation, scanning over a 2*θ* range of 20°–80° with 0.1° step size. The hydrodynamic sizes of the nanoparticles were measured using dynamic light scattering (DLS, Microtrac Zetatrac device, Model NPA152, maximum size range 6 μm), in DI water. The zeta potential was measured using a Microtrac Stabino device. The magnetic properties of the MNPs were characterized by a Physical Properties Measurement System (PPMS, Quantum Design) under magnetic fields of up to 15 kOe (= 150 mT). The nanoparticle core morphology was characterized by Transmission Electron Microscopy (TEM, Hitachi 7650 microscope). Nanoparticles’ surface morphology and elemental component analysis were performed using Scanning Electron Microscopy (SEM) and Energy-Dispersive X-ray (EDX) Spectroscopy (Zeiss Crossbeam 540 FIB-SEM attached with EDX), respectively.

### 2.3 | Cell Culture Preparation

SKOV3 ovarian cell line (ATCC, Rockville, MD) was maintained in Dulbecco’s Modified Eagle Medium (DMEM; Sigma–Aldrich, St. Louis, MO) supplemented with 10% fetal bovine serum (FBS; Corning, USA) and antibiotics (Corning, USA), under a controlled atmosphere of 5% CO_2_ at 37 °C. For experiments, cells were rinsed with phosphate-buffered saline (PBS, Sigma–Aldrich) and detached using 0.25% trypsin solution (Sigma–Aldrich, St. Louis, MO). The resulting suspension was centrifuged at 2000 rpm for 5 min to pellet the cells. The pellet was resuspended in fresh medium to obtain a final concentration of 4 × 10^5^ cells/mL, from which 1 mL was dispensed onto a petri dish for further use.

### 2.4 | Cell Viability and Colloidal Stability Test

Cell viability was assessed using a fluorescence-based Live/Dead assay (Biotium, Fremont, CA) following a 24 h incubation with 100 and 500 μg/mL MNPs at 37 °C and 5% CO_2_. To prepare the staining solution, Calcein AM and ethidium homodimer III were diluted in culture medium to the desired working concentrations. The treated cell samples were then exposed to this staining mixture for 20 min at 37 °C before imaging under a fluorescence microscope. After a 24 h exposure to 100 and 500 μg/mL MNPs, live cells exhibited green fluorescence (Calcein AM) with dead cells as red fluorescence (EthD-III). Furthermore, we have performed the colloidal stability test of 55 h milled nanoparticles with 2 mg/mL for up to 5 days.

### 2.5 | Magnetic Hyperthermia Performance Characterization

Magnetic hyperthermia experiments were conducted using the MagneTherm system (nanoTherics Ltd), equipped with water-cooled coils. For each measurement, the MNP powder was redispersed in 1 mL of DI water and mixed evenly in a sonicator (97043–996, VWR) for 5 min. Then, the mixture was inserted into the MagneTherm system. The precise temperature in the mixture was monitored using an optical fiber probe under both continuous and pulsed AMFs, as illustrated in Figure 1B. Based on the recorded temperature-time data, key performance metrics, including the SAR and ILP, were calculated using the following equations [38]:

(1) $$SAR=\frac{P}{m_{MNP}}$$

where the SAR (W/kg) for magnetic hyperthermia defines the heating power (*P*) generated per unit mass of MNP (*m*_*MNP*_), and *P* is defined as follows [14]:

(2) $$P=\pi \mu_{o}\chi_{0}H_{0}^{2}f\frac{2\pi f\tau }{1+(2\pi f\tau {)}^{2}}$$

where *f* and are the AMF frequency and amplitude, respectively, *τ* is the relaxation time of the MNPs, *μ*_*o*_ is the free space permittivity, and *χ*_0_ is the equilibrium susceptibility.

The SAR is calculated as follows [31]:

(3) $$SAR=\frac{Cp}{\varphi }\frac{\Delta T}{\Delta t}$$

where C_p_ (= 4,200 J/(Kg/K)) is the specific heat of water medium, Δ*T*/Δ*t* is the initial slope in the hyperthermia curve from 0 to 60 s, and *φ* is the mass of the MNPs per unit mass of the liquid. As for the SAR, the power also scales linearly with the AMF frequency (*f*) and quadratically with the AMF amplitude (*H*_0_). The ILP (nH m^2^/kg) removes these extrinsic factors and focuses on the MNPs’ heating performance, and it is defined as follows [38]:

(4) $$ILP=\frac{SAR}{f\times H^{2}}$$

The magnetic hyperthermia test was performed on various media (1 mL), including three different concentrations of PBS (10X, 5X, and 1X diluted in DI water), FBS, and agar gel. The agar gel was prepared by dissolving agar powder (Thermo Scientific, USA) in DI water at 2% (w/v) and heated on a hot plate with magnetic stirring to 90 °C–95 °C until optically clear (held 1–2 min), then cooled down to ~50 °C. Subsequently, 1 mL of the warm agar solution was combined with an aqueous MNPs suspension to reach the desired final volume, mixed gently to minimize air entrapment, transferred to a hyperthermia measurement vial, and allowed to form a fully gelled structure at 4 °C for 1 h before use.

## 3 | Results and Discussion

### 3.1 | Magnetocrystalline, Morphology, Size, and Magnetic Properties of MNPs

**FIGURE 2 |:**
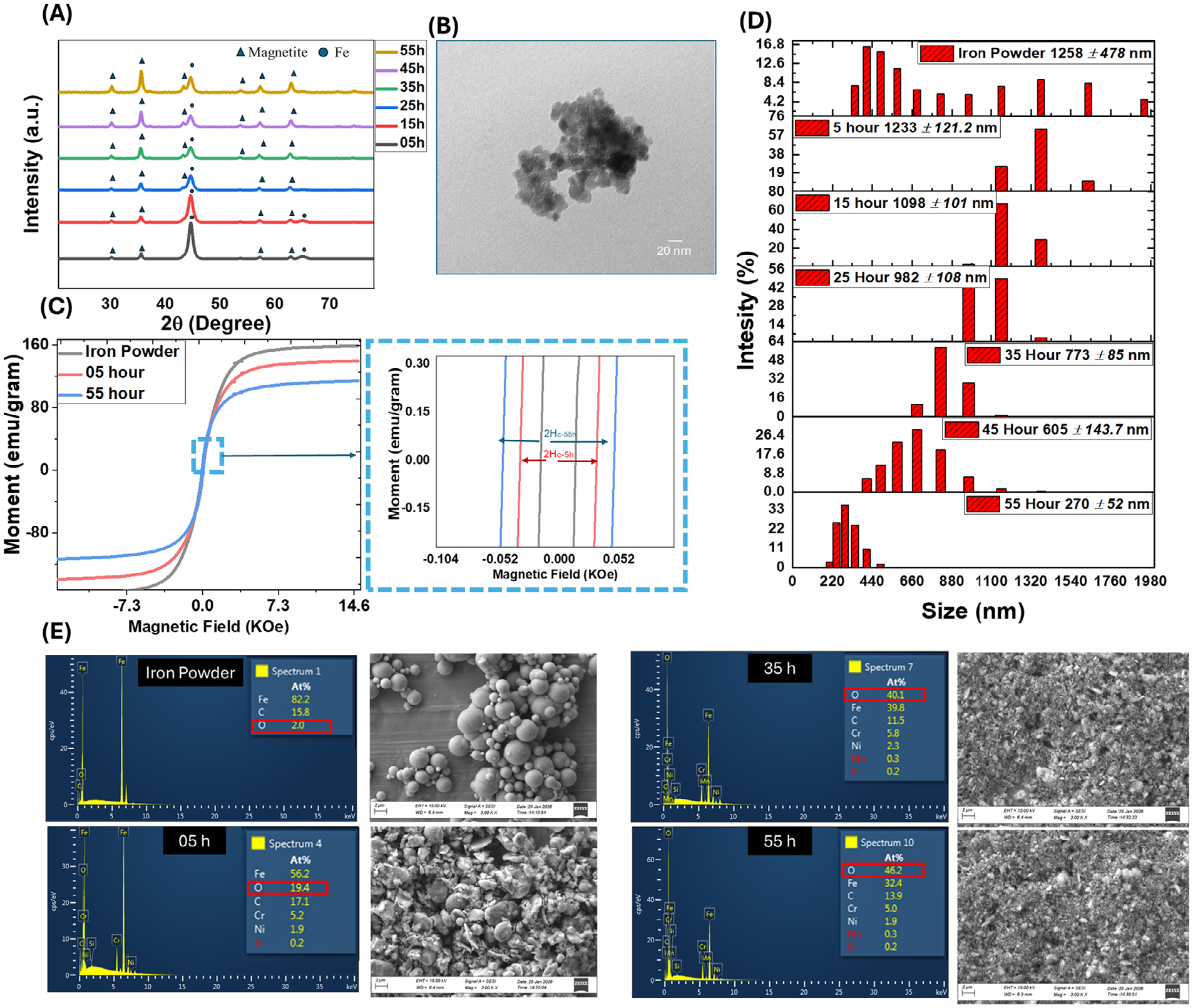
(A) XRD patterns of samples with varying milling times show a gradual decrease in the intensity of the precursor Fe peaks (marked with a circular label) with increased milling duration, indicating phase transformation from Fe to magnetite (marked with a triangle label). (B) TEM image of the nanoparticles after 55 h of milling. (C) The DC magnetization curves of the Iron powder, 5 and 55 h milled MNPs. The coercivities near zero fields are zoomed in. (D) DLS measurement of the hydrodynamic size of MNPs dispersed in DI water with the standard deviation from iron powder to 55 h milled particles. (E) SEM imaging and EDX spectroscopy of the iron powder and ball-milled nanoparticles.

The XRD patterns confirmed the crystallinity of the synthesized MNPs at various milling durations. As shown in Figure 2A, with increasing milling time from 5 to 55 h, the intensity of the Fe phase peaks gradually decreases, compared between 5 and 55 h milled samples, while the characteristic peaks of magnetite become more pronounced, indicating a phase transformation toward magnetite with longer milling time. Figure 2B shows a TEM image of the 55 h milled MNPs, where the irregular morphology and aggregations were observed. The irregular shapes of MNPs are due to the mechanical collisions of MNPs with stainless steel balls, which is a characteristic of ball-milled products. Due to the absence of surface coatings on these MNPs, along with non-zero remanence magnetizations (Figure 2C), these MNPs formed clusters that could be observed in the TEM images. The magnetic properties of dried powders were further characterized via PPMS, as shown in Figure 2C, where the Iron powder shows saturation magnetization (M_s_) around 162 emu/g and the hysteresis width around 29 Oe, the 5 h milled MNP sample exhibited a saturation magnetization (M_s_) of 139 emu/g and a hysteresis width (2H_c_) of ~66 Oe, whereas the 55 h milled sample exhibited a saturation magnetization of 114 emu/g and a hysteresis width (2H_c_) of ~98 Oe. The increase in coercivity with the increased milling time indicates the dominant ferromagnetic behavior at room temperature [39], and these nanoparticles are in the multidomain regions [40]. Moreover, bulk Fe exhibits a theoretical M_s_ of ~220 emu/g, while bulk Fe_3_O_4_ has a theoretical M_s_ of ~90 emu/g. As with longer milling times, the MNP size decreases, surface spin-canting effects become more prominent, resulting in a reduction in M_s_. Additionally, prolonged ball milling increases the percentage of the Fe_3_O_4_ phase, further contributing to the observed decrease in M_s_ in 55–5 h millied MNPs.When dispersed in DI water, the Fe surface develops hydroxyl (−OH) surface chemical groups; thus, the zeta potential results show a surface charge of −60 mV. The DLS results in Figure 2D indicate that the hydrodynamic size of these ball-milled MNPs decreases with milling time from 5 to 55 h, and also the standard deviation data show that the 55 h milled samples show a smaller size distribution compared to the 5 h milled samples. The 55 h milled nanoparticle has an average hydrodynamic size of 270 nm with a standard deviation of 52 nm, while the 5 h nanoparticle has an average hydrodynamic size of 1233 nm and a standard deviation of 121.3 nm. It can also be observed from the surface morphology imaging of SEM that from Iron powder to 55 h milled nanoparticles size decreased significantly, as shown in Figure 2E. Additionally, the EDX results shown in Figure 2E confirm that more oxygen is incorporated due to the transformation of iron powder to the Fe_3_O_4_ structure as the milling time increases, such as 5, 35, and 55 h. EDX measurements show oxygen percentages of 19.4%, 40.1%, and 46.2%, respectively. Also, due to grinding in the stainless steel jar and balls, there are small amounts of impurities (Cr & Ni) observed in the elemental mapping.

### 3.2 | Magnetic Hyperthermia Performance of MNPs

**FIGURE 3 |:**
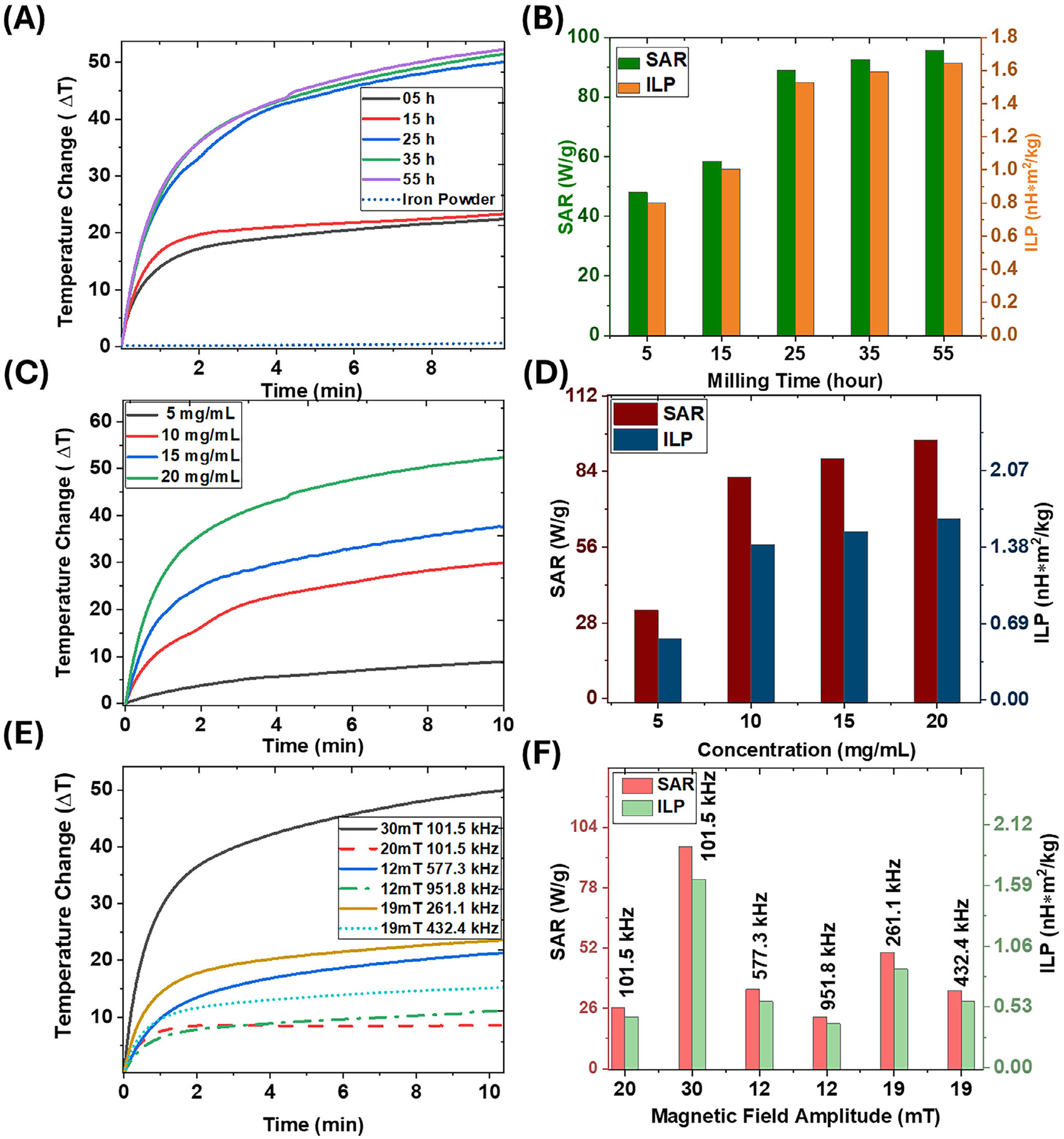
(A) Magnetic hyperthermia measurements using the calorimetric method for MNPs milled for different durations (5–55 h milled) under an AMF of 30 mT and 101.5 kHz. The MNPs were dispersed in DI water to reach a 20 mg/mL concentration. Hyperthermia results show improved heating efficiency of MNPs synthesized at longer milling times. (B) SAR and ILP values from MNPs prepared under different milling durations. (C) Temperature rise of the 55 h milled MNPs at different concentrations (5–20 mg/mL) under an AMF of 30 mT and 101.5 kHz, demonstrating that heat generation increases with MNP concentration. (D) SAR and ILP values from MNPs prepared under different concentrations. (E) Magnetic hyperthermia measurements for the 55 h milled MNPs at a 20 mg/mL concentration under varying AMF profiles. The results demonstrate that higher field amplitudes and lower frequencies lead to greater heat generation. (F) SAR and ILP values from MNPs under various magnetic field amplitudes and frequencies.

We began our tests by evaluating first the hyperthermia performance of ball-milled nanoparticles synthesized at different milling durations: 5, 15, 25, 35, and 55 h milled, while maintaining a constant particle concentration of 20 mg/mL in DI water and an AMF of 30 mT at 101.5 kHz. Figure 3A shows that the precursor Fe powders (also dispersed in DI water to arrive at the established 20 mg/mL concentration) exhibited negligible heat generation, whereas a noticeable temperature rise was observed only after the particles began transitioning to a magnetite-like composition as a result of the ball milling process. The temperature rise (*ΔT*) for the 5 and 15 h milled MNPs was relatively modest, reaching a maximum of ~23 °C. In contrast, MNPs milled for 25, 35, and 55 h showed significantly enhanced heating, with *ΔT* values of around 50 °C, 51 °C, and 52 °C, respectively. These results indicate that extended milling improves magnetic heating efficiency up to a threshold, beyond which the performance stabilizes. The heating efficiency of the MNPs improved with extended milling time, primarily due to the increased hysteresis width from 66 Oe (5 h milled) to 98 Oe (55 h milled), as illustrated in Figure 2C. This observation of hysteresis width enhancement was further supported by our XRD analysis in Figure 2A, which revealed that with longer milling times, MNPs were becoming magnetite, while the Fe phase was observed to be reduced for longer synthesis processes. Corresponding SAR and ILP values calculated from Equations (3) and (4) are summarized in Figure 3B, where we observed a remarkable increase starting at 25 h milled, with values remaining relatively stable thereafter.

Additionally, we varied the 55 h milled MNP concentrations (5, 10, 15, and 20 mg/mL) to evaluate the effect of nanoparticle concentration on hyperthermia performance, under the same AMF condition (30 mT, 101.5 kHz). As illustrated in Figure 3C, the temperature increase was positively correlated with particle concentration, yielding maximum *ΔT* values of ~9 °C, 30 °C, 37 °C, and 52 °C, for 5, 10, 15, and 20 mg/mL concentrations, respectively. The corresponding SAR and ILP values, shown in Figure 3D, were initially low at 5 mg/mL (SAR ~32 W/g and ILP ~0.55 nH m^2^/kg), but exhibited a steady increase when the concentration rose from 10 mg/mL (SAR ~ 82 W/g and ILP ~ 1.4 nH ⋅m^2^/kg) to 20 mg/mL (SAR ~95 W/g and ILP ~1.6 nH m^2^/kg), highlighting the enhanced heating performance at higher concentrations. SAR and ILP increase with concentration because, under the same excitations, the initial heat generation slope is significantly higher at 10 than at 5 mg/mL concentrations. As the SAR is directly related to the initial slope of heating, and ILP is proportional to SAR (Equations 3 and 4), the ILP also changes drastically with concentration, initially from 5 to 10 mg/mL and remaining steady from 10 to 20 mg/mL. At a fixed medium volume of 1 mL of DI water, increasing the MNP mass or concentration results in greater heat generation.

### 3.3 | Field Effect on MNPs’ Hyperthermia Performance

We further investigated the effects of varying the amplitude and frequency of the AMF on the magnetic hyperthermia performance of the 55 h milled MNPs. Initially, we varied the field amplitude (20 and 30 mT) while keeping the frequency at 101.5 kHz. As shown in Figure 3E, higher AMF amplitude resulted in significantly enhanced heating, with the maximum temperature increase (*ΔT*) rising from ~8 °C at 20 mT to ~50 °C at 30 mT. Correspondingly, the SAR and ILP values, presented in Figure 3F, also increased, with values rising from 26 W/g and 0.44 nH m^2^/kg at 20 mT, to 95 W/g and 1.6 nH m^2^/kg at 30 mT, respectively. As the power produced by the MNPs is directly related to the amplitude of the magnetic field (Equation (2)), increasing the magnetic field amplitude from 20 to 30 mT enhanced the MNPs’ heating and SAR significantly. Also, despite the effect of the AMF amplitude and frequency, the MNPs generated additional heat, which can be confirmed by an increase in the ILP values.

We then examined the effect of AMF frequency at two fixed field amplitudes of 12 and 19 mT. At 12 mT, frequencies of 577.3 and 951.8 kHz were applied, and at 19 mT, frequencies of 261.1 and 432.4 kHz were provided. The results, shown in Figure 3E, indicate that the temperature rise dropped with higher AMF frequency values, which is because MNPs’ magnetizations cannot follow the field as frequency increases. For example, at 12 mT, the *ΔT* decreased from ~21 °C (577.3 kHz) to ~11 °C (951.8 kHz), while at 19 mT, it declined from ~23 °C (261.1 kHz) to ~15 °C (432.4 kHz). The corresponding SAR and ILP values (Figure 3F) justify our observation that the heating performance decreased with increasing AMF frequency. Specifically, SAR and ILP dropped from 34 W/g and 0.57 nH ·m^2^/kg to 22 W/g and 0.38 nH m^2^/kg for 12 mT as we increased frequency from 577.3 kHz to 951.8 kHz. Similarly, the SAR and 0.57 nH m^2^/kg for 19 mT as we increased frequency from 261.1 to 432.4 kHz.

### 3.4 | Temperature Regulation via Pulsed AMF

**FIGURE 4 |:**
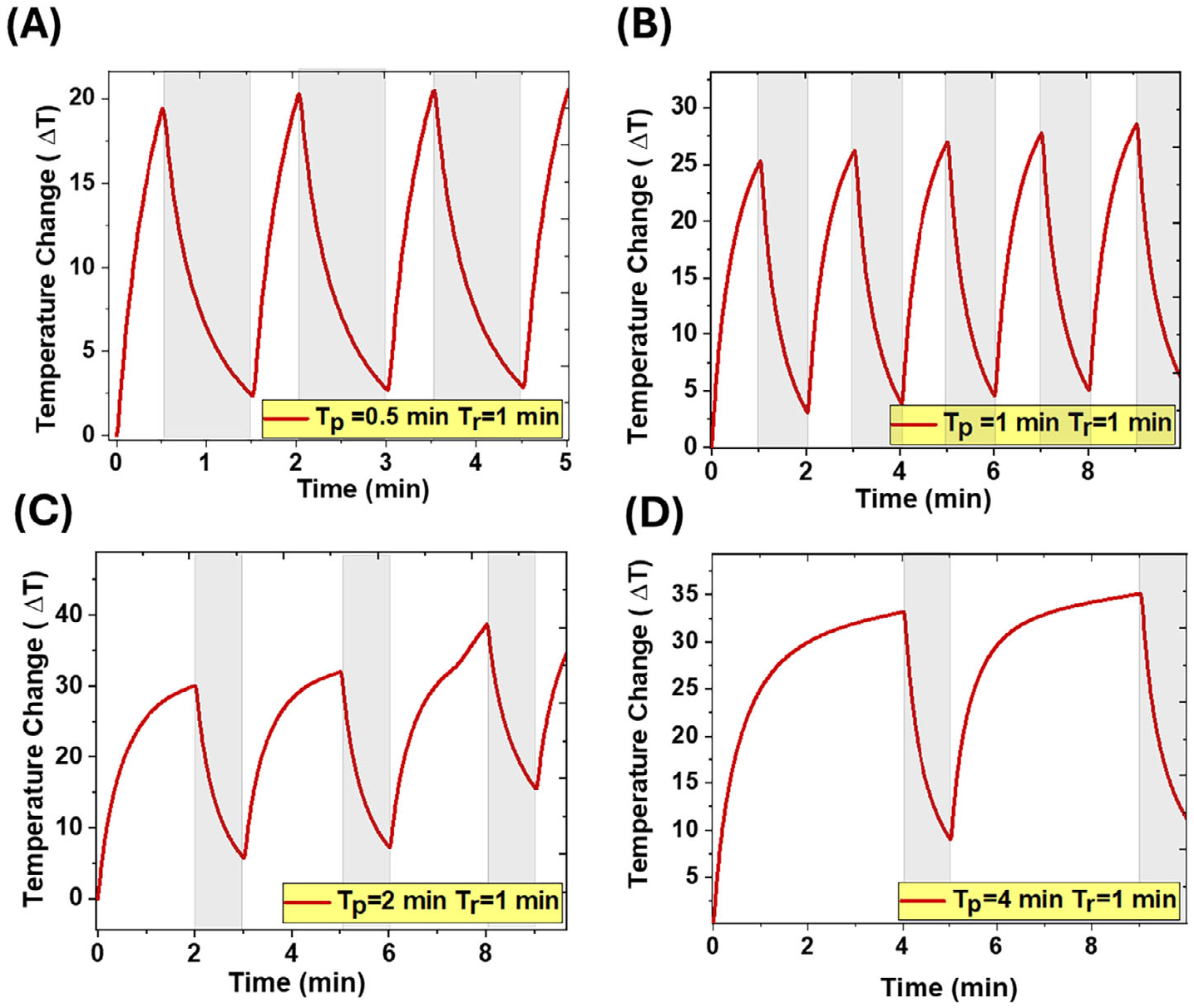
Controlled magnetic hyperthermia heating using pulsed AMFs with varying excitation pulse widths of (A) T_p_ = 0.5 min, (B) T_p_ = 1 min, (C) T_p_ = 2 min, and (D) T_p_ = 4 min. The relaxation time T_r_ was fixed at 1 min for all scenarios.

To explore the feasibility of temperature regulation during hyperthermia treatment, we applied pulsed AMFs with varying excitation pulse widths (T_p_ = 0.5, 1, 2, and 4 min) while keeping the relaxation pulse width (T_r_) constant at 1 min. As illustrated in Figure 4A–D, pulsed excitations enabled controlled heating of the 55 h milled MNPs. The maximum temperature increase was limited to ~20 °C, 25 °C, 30 °C, and 35 °C for T_p_ values of 0.5, 1, 2, and 4 min, respectively, demonstrating a promising method for heat modulation in magnetic hyperthermia applications. The gray areas in Figure 4 indicate the periods when the AMF was turned OFF, also known as relaxation time, showcasing a sharp drop in the temperature. Moreover, we can also observe that pulsing induces semi-adiabatic temperature growth at each pulse’s starting point. Depending on the excitation pulse widths, we can control the overall heating as well as the semi-adiabatic temperature growth after each pulse.

### 3.5 | Medium Effect on MNPs’ Hyperthermia Performance

The PPMS measurements revealed that the hysteresis width (2H_c_) of our MNPs was approximately 60–98 Oe as we increased milling time from 5 to 55 h, which indicates that these MNPs are not superparamagnetic. Due to their relatively large size and clustering in the solution medium, the heat generation is primarily attributed to hysteresis losses. As the hysteresis loop mainly governs heat generation in MNPs [32], the heating behavior differs depending on the magnetic regime. For instance, for superparamagnetic MNPs, magnetic losses generally increase with frequency due to enhanced relaxation dynamics. In contrast, for larger ferromagnetic nanoparticles, the heating is governed dominantly by hysteresis losses, and the field amplitude has a more significant impact on heat generation than frequency [41].

**FIGURE 5 |:**
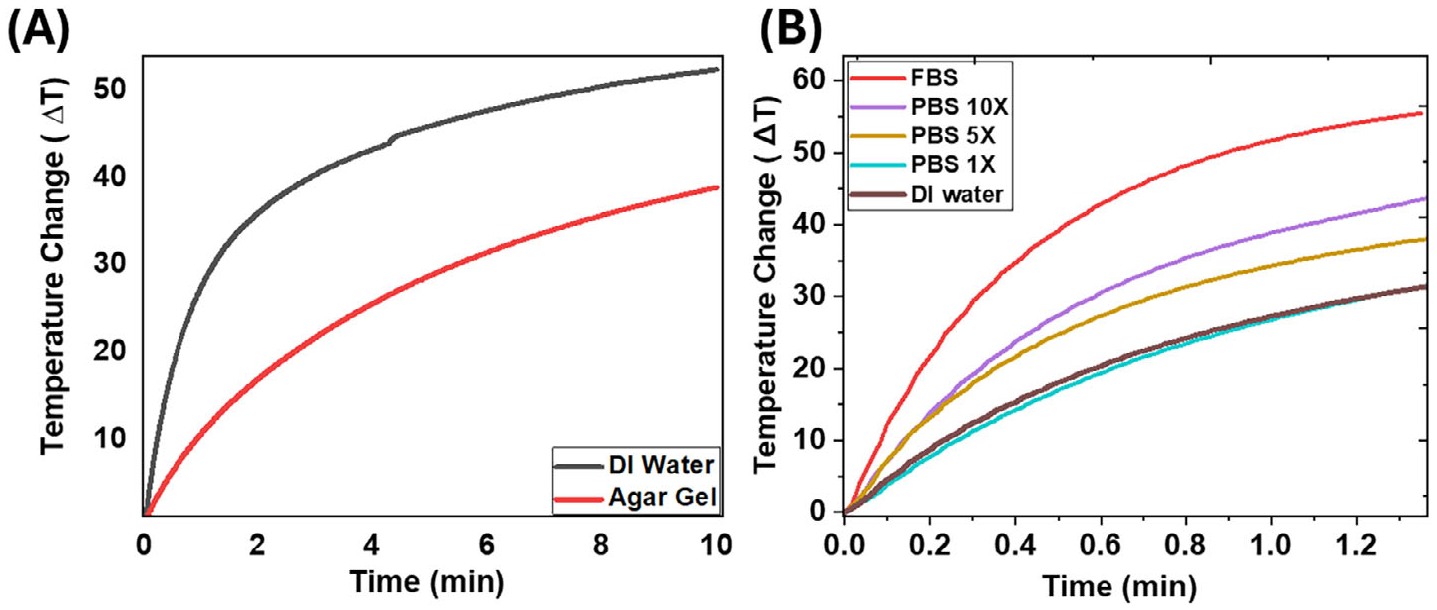
Effect of media composition on the hyperthermia performance of MNPs. (A) The *T–t* curves of 20 mg/mL, 55 h milled MNPs when dispersed in DI water (black curve) and agar gel (red curve). Brownian relaxation was blocked in agar gel. (B) The *T–t* curves of 20 mg/mL, 55 h milled MNPs dispersed in DI water, FBS, 1X, 5X, and 10X PBS. All characterizations were done under an AMF of 30 mT and 101.5 kHz.

To demonstrate that the heat was dominantly generated from hysteresis losses in our material, we used agar gel to block the Brownian relaxation and performed the hyperthermia measurements depicted in Figure 5A. This confirmed that the 55 h milled MNPs could generate heat even without any Brownian relaxation, and the heat was mostly coming from the coercivity and Néel relaxations. It also explains why the 5 h milled MNPs generated less heat than the 55 h milled MNPs, as seen in Figure 3A. Also, the agar gel is considered a tumor-mimicking environment, so the fact that our synthesized MNPs are suitable for generating heat in this medium is highly beneficial for their intended use in in vivo applications for cancer therapy.

We further tested the heating performance of MNPs dispersed in different media. From Figure 5B, we can observe that the heating effect varied based on the medium concentration of the PBS (1X, 5X, and 10X) and FBS, with ΔT of 30 °C, 35 °C, 40 °C, and 55 °C, respectively, for an excitation time of 1.4 min. The slope of the temperature curve varied as we modified the media. For higher PBS concentrations (lower dilution factors), higher heating was obtained, and similarly, for the FBS medium, a higher temperature change slope was seen. The specific heat of the PBS decreases as we increase the PBS concentration [42] from 1X to 10X; therefore, the temperature change slope for the PBS with increasing the concentration from 1X to 5X and to 10X showed a higher temperature change trend. For the FBS medium, we hypothesize that the specific heat of FBS is lower than that of 10X PBS; therefore, the temperature change slope is higher for this material compared to DI water and PBS.

### 3.6 | Cellular Uptake, Biocompatibility, Colloidal Stability, and Hyperthermia-Induced Apoptosis of Ball-Milled MNPs

**FIGURE 6 |:**
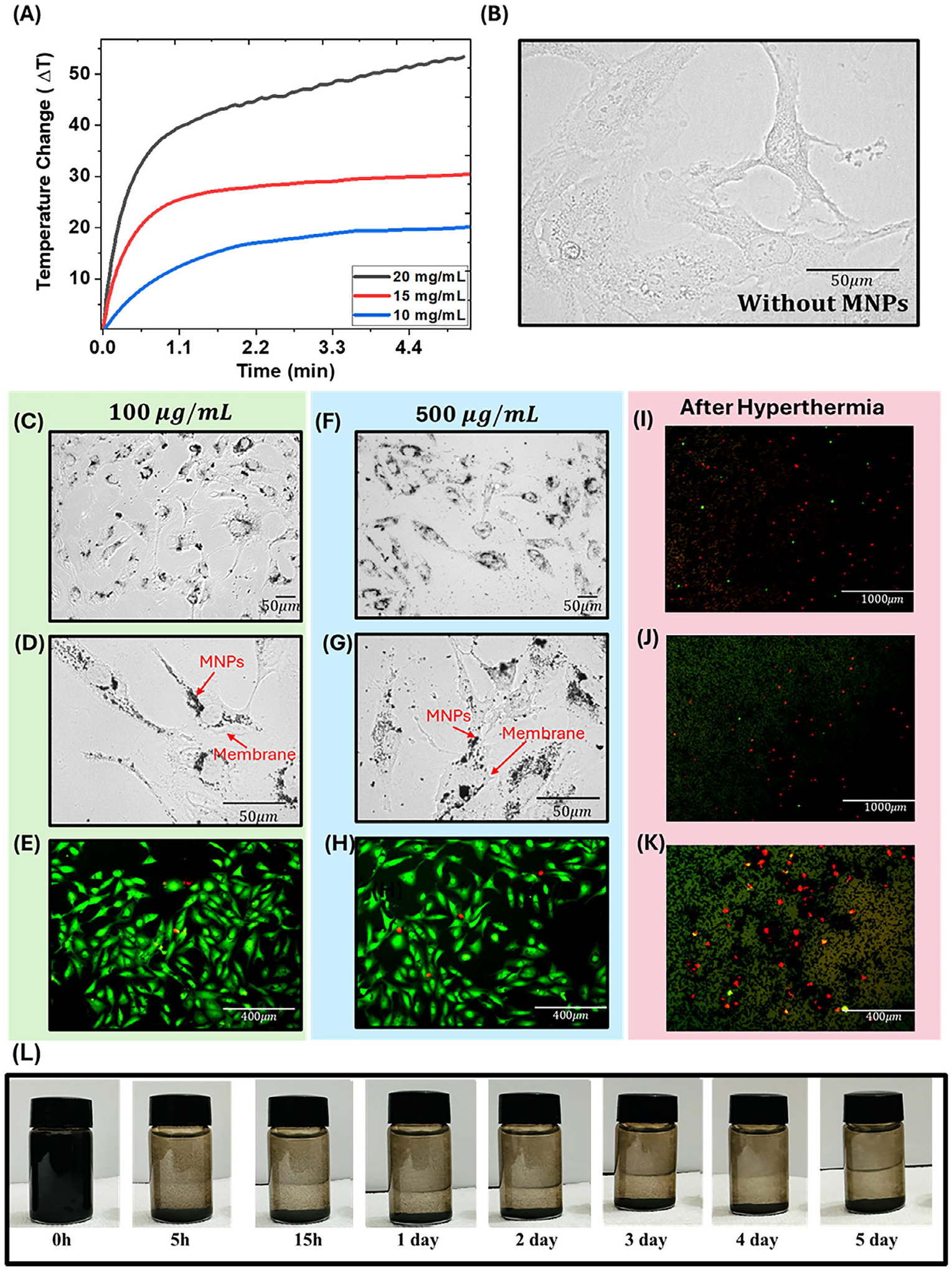
(A) Magnetic hyperthermia measurements of our 55 h milled MNPs at 10, 15, and 20 mg/mL concentration after 1 h incubation in SKOV3 ovarian cancer cells. (B) Control SKOV3 cells without MNPs. (C) SKOV3 cells after a 24 h incubation with 100 μg/mL MNPs. (D) Zoomed view of (C) showing the cellular membrane and internalized MNPs. (E) Live/Dead assay for cells treated with 100 μg/mL MNPs: viable cells appear in green, dead cells appear in red. (F) SKOV3 cells after a 24 h incubation with 500 μg/mL MNPs. (G) Zoomed view of (F) showing the cellular membrane and internalized MNPs. (H) Live/Dead assay for cells treated with 500 μg/mL MNPs. (I–J) Live/Dead fluorescence images of SKOV3 cells after magnetic hyperthermia treatment at different regions of the microscope field of view, and (K) a magnified view, showing predominantly dead cells. (L) Coloidal stability test for 55 h milled MNPs from 5 h to 5 days with a concentration of 2 mg/mL.

The magnetic hyperthermia performance of our 55 h milled MNPs was evaluated using SKOV3 ovarian cancer cells incubated with 10, 15, and 20 mg/mL of MNPs for 1 h at 37 °C and 5% CO_2_. Under an AMF of 30 mT and 101.5 kHz, temperature rises of ~20 °C, 30 °C, and 55 °C were observed, respectively, as presented in Figure 6A, which was consistent with the concentration-dependent trends previously presented in Figure 3C. Cellular uptake was confirmed following a 24 h incubation with 100 and 500 μg/mL of MNPs. Microscopy images in Figure 6C–G showed extensive internalization and effective cellular uptake when compared to control cells without MNPs (Figure 6B). Further, cell viability after 24 h incubation with 100 and 500 μg/mL MNPs in SKOV3 cells was assessed using Live/Dead fluorescence staining, which showed predominantly green fluorescence with negligible red signals, thereby confirming high viability and minimal cytotoxicity (Figure 6E,H). To assess hyperthermia-induced cell death, SKOV3 cells treated with 20 mg/mL MNPs were exposed to AMF (30 mT, 101.5 kHz) and stained with Live/Dead dyes. Imaging revealed predominantly red fluorescence (Figure 6I–K), confirming efficient hyperthermia-induced apoptosis. Cellular uptake and biocompatibility are often tested at lower concentrations in vitro (lab dish) for testing the intrinsic cytotoxicity and cellular uptake of the nanomaterials [37, 43]. Collectively, these results demonstrate robust cellular uptake, excellent biocompatibility under physiological conditions, and potent therapeutic efficacy upon AMF excitation. Figure 6L demonstrates the colloidal stability of the 55 h milled MNPs over a period of up to 5 days. Most MNPs precipitated within the first 5 h; however, despite being uncoated and having a relatively large hydrodynamic size, a fraction remained stably suspended in deionized water for up to 5 days.

## 4 | Conclusions

This study provides a comprehensive investigation of MNPs synthesized through mechanochemical high-energy wet ball milling, systematically exploring their potential for magnetic hyperthermia applications. By varying the milling time, we demonstrated that prolonged milling reduces the size of pristine Fe particles while promoting a phase transition from Fe to magnetite. This structural and size transition directly enhanced the heating efficiency of the ball-milled MNPs by widening their magnetic hysteresis loops, thereby improving their capacity to generate heat under AMFs. Under clinically relevant AMF conditions of 30 mT at 101.5 kHz, the 55 h milled MNPs achieved a notable temperature rise (*ΔT*) of ~50 °C within 10 min, with corresponding SAR and ILP values of 95.57 W/g and 1.64 nH·m^2^/kg, respectively. Importantly, these performance metrics fall within safety limits recommended for human use, underscoring the translational relevance of the ball-milled particles.

In addition to the effect of milling time, we also reported that heating efficiency strongly depends on particle concentration and AMF amplitude, while higher excitation frequencies reduce performance due to a diminished magnetic response. Beyond conventional continuous-wave excitation, we investigated the potential of pulsed AMF excitation as a strategy for thermal modulation. By modulating pulse-width ratio, we demonstrated that semi-adiabatic heating could be achieved, enabling more precise control over temperature rise and potentially reducing risks of thermal damage to healthy tissues. This capability for programmable thermal dosing adds an important layer of safety and tunability to hyperthermia therapies based on ball-milled MNPs. The impact of biological media on hyperthermia performance was further assessed by dispersing the 55 h milled MNPs in agar gel, PBS, and FBS. Across all conditions, the MNPs retained robust heating capability, indicating that their magnetic and thermal properties are stable even in tumor-mimicking and physiologically relevant environments. Such resilience is critical for advancing MNPs beyond in vitro settings toward preclinical and clinical testing. Complementary cellular studies with SKOV3 ovarian cancer cells confirmed effective uptake of the MNPs and negligible cytotoxicity at concentrations up to 500 μg/mL, demonstrating that the ball-milled MNPs are well tolerated by living cells. Following magnetic hyperthermia treatment, Live/Dead assays revealed substantial cancer cell death, thereby validating both the therapeutic efficacy and biosafety of these materials.

This work highlights the strong promise of ball-milled MNPs as efficient, controllable, and biocompatible agents for magnetic hyperthermia. The combination of high heating performance, stability across diverse biological environments, programmable thermal control, and favorable cellular interactions provides a multifaceted platform for next-generation cancer therapy. Future research should focus on refining the mechanochemical synthesis conditions to further optimize particle size distribution, phase composition, and magnetic anisotropy. Equally important will be the development of surface functionalization strategies that enhance colloidal stability in physiological fluids, extend circulation time in vivo, and enable tumor-specific targeting. Integration of these advances with systematic in vivo studies will be essential for bridging the gap from benchtop demonstrations to clinically viable hyperthermia treatments.

## Data Availability

The data that support the findings of this study are available from the corresponding author upon reasonable request.
